# Supplementary material for: Phylogenomics reveals the evolutionary origins of lichenization in chlorophyte algae
Source: Nat Commun. 2024 May 24;15:4452. doi: 10.1038/s41467-024-48787-z (PMC11126685; doi:10.1038/s41467-024-48787-z)
Supplement: Supplementary file 15 — Reporting Summary [file 41467_2024_48787_MOESM15_ESM.pdf]

nature portfolio

Corresponding author(s): Pierre-Marc Delaux
Last updated by author(s): 19/04/2024

Reporting Summary

Nature Portfolio wishes to improve the reproducibility of the work that we publish. This form provides structure for consistency and transparency in reporting. For further information on Nature Portfolio policies, see our Editorial Policies and the Editorial Policy Checklist.

Statistics

For all statistical analyses, confirm that the following items are present in the figure legend, table legend, main text, or Methods section.

n/a	Confirmed	
☐	☒	The exact sample size (*n*) for each experimental group/condition, given as a discrete number and unit of measurement
☐	☒	A statement on whether measurements were taken from distinct samples or whether the same sample was measured repeatedly
☐	☒	The statistical test(s) used AND whether they are one- or two-sided<br>*Only common tests should be described solely by name; describe more complex techniques in the Methods section.*
☒	☐	A description of all covariates tested
☒	☐	A description of any assumptions or corrections, such as tests of normality and adjustment for multiple comparisons
☐	☒	A full description of the statistical parameters including central tendency (e.g. means) or other basic estimates (e.g. regression coefficient) AND variation (e.g. standard deviation) or associated estimates of uncertainty (e.g. confidence intervals)
☐	☒	For null hypothesis testing, the test statistic (e.g. *F*, *t*, *r*) with confidence intervals, effect sizes, degrees of freedom and *P* value noted<br>*Give P values as exact values whenever suitable.*
☒	☐	For Bayesian analysis, information on the choice of priors and Markov chain Monte Carlo settings
☐	☒	For hierarchical and complex designs, identification of the appropriate level for tests and full reporting of outcomes
☒	☐	Estimates of effect sizes (e.g. Cohen's *d*, Pearson's *r*), indicating how they were calculated

Our web collection on statistics for biologists contains articles on many of the points above.

Software and code

Policy information about availability of computer code

Data collection	No software was used
Data analysis	lima 1.9.0; Flye 2.7-b1587; GCpp 2.0.0; SSPACE-LongRead 1.1; MEGAN 6.6.7; BRAKER2; cutadapt 2.1; TrimGalore 0.6.5; HISAT2 2.1.0; samtools 1.10; DRAP 1.92; TransDecoder 5.5.0; InterProScan 5.48-83.0; OrthoFinder 2.5.4; R 4.2.2; ape 5.7-1; phytools 1.9.16; EDTA 2.0.1; LTR_FINDER 1.07; LTR_retriever 2.6; TIR-Learner 1.19; HelitronScanner 1.1 Generic Repeat Finder 1.0; RepeatModeler 2.0.3; TESorter; RepeatMasker 4.1.2-p1; mixOmics 6.22.0; BLASTp+ 2.9.0; MUSCLE 5.1.0; trimAl 1.4.1; IQTREE 2.1.3; NFCORE/RNASEQ 3.4; nextflow 21.04; bedtools 2.30.0; cutadapt 3.4; TrimGalore 0.6.7; picard 2.25.7; salmon 1.5.2; samtools 1.13; star 2.6.1d; star 2.7.6a; stringtie 2.1.7; UCSC tools 377; edgeR 2.1.7; R 4.1.1; chromoMap 0.3.1; minimap2 2.17-r941; BLASTp+ 2.13.0.1; FastTree 2.1.10; HMMER 3.3.1; AlphaFold 2.1.0; DIAMOND 0.9.19; ModelFinder; iTOL 6; R v4.1.1; CUPP 4.0.0

For manuscripts utilizing custom algorithms or software that are central to the research but not yet described in published literature, software must be made available to editors and reviewers. We strongly encourage code deposition in a community repository (e.g. GitHub). See the Nature Portfolio guidelines for submitting code & software for further information.

Data

Policy information about availability of data

All manuscripts must include a data availability statement. This statement should provide the following information, where applicable:

- Accession codes, unique identifiers, or web links for publicly available datasets
- A description of any restrictions on data availability
- For clinical datasets or third party data, please ensure that the statement adheres to our policy

Genome and transcriptome data from this study were deposited in NCBI under the BioProject PRJNA790449.
The following database were used in this study: NCBI NR (accessed September 2020 for genome annotation and May 2022 for HGT investigation), Pfam v32 (1KP transcriptome annotation) and v34 (this study transcriptome annotation), OrthoDB v10, Mycocosm (last accessed in February 2020) and SwissProt (last accessed September 2021 for transcriptome annotation from this study and January 2019 for transcriptome annotation from the 1KP project).
Publicly available genomes used in this study can be found in the NCBI under the following accession codes: Auxenochlorella protothecoides 710 [GCF_000733215.1], Auxenochlorella protothecoides UTEX25 [GCA_003709365.1], Chlorella sorokiniana [GCA_002245835.2], Chlorella variabilis NC64A [GCF_000147415.1], Helicosporidium sp. ATCC 50920 [GCA_000690575.1], Micractinium conductrix SAG241.80 [GCA_002245815.2], Parachlorella kessleri iCABeR21 [GCA_015712045.1], Prototheca wickerhamii HMC1 [GCA_003255715.1], Ulva prolifera [GCA_004138255.1].
Picochlorum sp. RCC4223 and Ulva mutabilis genomes were retrieved from ORCAE database respectively at https://bioinformatics.psb.ugent.be/gdb/RCC4223/ and https://bioinformatics.psb.ugent.be/gdb/ulva/.
Transcriptomes of Pseudochlorella pringsheimii, Watanabea reniformis, Elliptochloris marina were assembled from SRA available in the NCBI under the respective series of SRA accession codes: [SRR11611235, SRR11611236, SRR11611237, SRR11611238], [SRR16849198] and [SRR3952294, SRR5133332] respectively.
Annotations of the 1KP transcriptomes have been deposited in FigShare under the DOI: 10.6084/m9.figshare.25611138

Research involving human participants, their data, or biological material

Policy information about studies with human participants or human data. See also policy information about sex, gender (identity/presentation), and sexual orientation and race, ethnicity and racism.

Reporting on sex and gender	The research did not involve human participants
Reporting on race, ethnicity, or other socially relevant groupings	The research did not involve human participants
Population characteristics	The research did not involve human participants
Recruitment	The research did not involve human participants
Ethics oversight	The research did not involve human participants

Note that full information on the approval of the study protocol must also be provided in the manuscript.

Field-specific reporting

Please select the one below that is the best fit for your research. If you are not sure, read the appropriate sections before making your selection.

☐ Life sciences ☐ Behavioural & social sciences ☒ Ecological, evolutionary & environmental sciences

For a reference copy of the document with all sections, see nature.com/documents/nr-reporting-summary-flat.pdf

Ecological, evolutionary & environmental sciences study design

All studies must disclose on these points even when the disclosure is negative.

Study description	The aim of the study was to decipher the evolutioanry of lichen-forming ability in chlorophyte algae using a combination of phylogenomic and transcriptomic approaches
Research sample	We used 26 and 103 publicly available genomes and transcriptomes covering the main classes and families of chlorophytes as well as including lichen-forming and non-lichen forming species. Genome data were obtain from the NCBI and transcriptomes from the 1KP project. To ensure a relevant coverage of lichen- close relative non-lichen forming species, we sequenced nine genomes and three transcriptomes. The total sampling includes 35 genomes and 106 transcriptomes covering all the main families of chlorophyte algae as well as 15 lichen-forming and 126 non-lichen-forming species. This unprecedent sampling fulfill the requirements for large-scale unsupervised phylogenomic approaches. We made all data publicly available through NCBI and FigShare (see data availability statement). Briefly, algae have been isolated using a micromanipulator and cultures grown on solid 3N BBM + V medium (Bold's Basal Medium with vitamins and triple nitrate71) under a 30 µmol/m2/s photosynthetic photon flux density with a 12 h photoperiod at 16° C, as described in the material and method section of the manuscript.
Sampling strategy	Three Trebouxia algae have been isolated from lichens with various ecology. They cover different adaptation to environment and

	have been cultivated on dedicated medium before being identified by comparing marker sequences to public database. For phylogenomic studies, only one representative per species is sufficient as we are not interested in population level comparison but only inter-species comparison. Thus the sampling is perfectly adequate for the scientific purpose covered in this study.
Data collection	Public genomes and transcriptomes were obtain from the NCBI and ORCAE database, the newly sequenced species have been obtained from the SAG database. Trebouxia sp. OTU1, 5 and 8 have been isolated from thalli of lichen Umbilicaria pustulata following the standard procedure described in Beck, A. & Hans-Ulrich, K. Analysis of the Photobiont Population in Lichens Using a Single-Cell Manipulator. Symbiosis 57–67 (2001).<br>J.K, C.P and C.L searched and downloaded the publicly available genomic and transcriptomic data. F.D.G isolated the algae from lichens and sequenced them. Isolation of algae from the lichen thallus is described in the method section of the manuscript.
Timing and spatial scale	This project does not involved timing nor spatial scale
Data exclusions	No data were excluded
Reproducibility	The in vitro enzymatic test with purified GH8 were repeaseted and revealed the same activity across 2 pH range and 3 replicates.
Randomization	Our study consists in genome comparison within an evolutionry frame, thus randomization is not applicable.
Blinding	This project aims to identify genes associated to lichen-forming capacity by comparing genomes in an evolutionary perspective according to the lichenization trait. Thus, blinding is not relevant in this case.

Did the study involve field work? ☐ Yes ☒ No

Reporting for specific materials, systems and methods

We require information from authors about some types of materials, experimental systems and methods used in many studies. Here, indicate whether each material, system or method listed is relevant to your study. If you are not sure if a list item applies to your research, read the appropriate section before selecting a response.

Materials & experimental systems

n/a	Involved in the study
☒	☐ Antibodies
☒	☐ Eukaryotic cell lines
☒	☐ Palaeontology and archaeology
☒	☐ Animals and other organisms
☒	☐ Clinical data
☒	☐ Dual use research of concern
☐	☒ Plants

Methods

n/a	Involved in the study
☒	☐ ChIP-seq
☒	☐ Flow cytometry
☒	☐ MRI-based neuroimaging

Dual use research of concern

Policy information about dual use research of concern

Hazards

Could the accidental, deliberate or reckless misuse of agents or technologies generated in the work, or the application of information presented in the manuscript, pose a threat to:

No	Yes	
☒	☐	Public health
☒	☐	National security
☒	☐	Crops and/or livestock
☒	☐	Ecosystems
☒	☐	Any other significant area

Experiments of concern

Does the work involve any of these experiments of concern:

No	Yes	
☒	☐	Demonstrate how to render a vaccine ineffective
☒	☐	Confer resistance to therapeutically useful antibiotics or antiviral agents
☒	☐	Enhance the virulence of a pathogen or render a nonpathogen virulent
☒	☐	Increase transmissibility of a pathogen
☒	☐	Alter the host range of a pathogen
☒	☐	Enable evasion of diagnostic/detection modalities
☒	☐	Enable the weaponization of a biological agent or toxin
☒	☐	Any other potentially harmful combination of experiments and agents

Plants

Seed stocks	Apatococcus fuscideae: SAG2523; Apatococcus lobatus: SAG2145; Coccomyxa pringsheimii SAG2167; Elliptochloris bilobata: SAG24580; Myrmecia biatorellae: SAG882; Myrmecia bisecta: SAG2043; Symbiochloris irregularis: SAG2036; Paulbroadya petersii: SAG2240; Paulbroadya prostrata: SAG2392; Trebouxia were obtained from Umbilicaria pustalata lichen from the LOEWE Centre for Translational Biodiversity Genomics
Novel plant genotypes	No novel plant genotypes have been produced in this project
Authentication	Any mutation has been generated in this project
